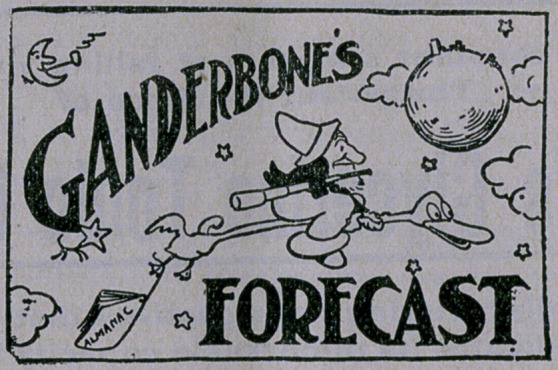# Abstracts and Selections

**Published:** 1908-10

**Authors:** 


### Abstracts and Selections.

**For October.**

(Copyright 1908, by C. H. Rieth.)

Now, Taft was enjoying
An aeroplane flight,
And skimming around
Like the man-swallow Wright,
When who should appear,
Holding fast to his hat,
And sailing the sky,
But the man from the Platte.

Said fat Bill to Platte Bill,
"Now, isn't this great?"
Said Bryan, "You bet—
Is my rudder on straight?"
And the wind coming up
With a slight show of bluster,
They both skidded off
Through the air belly buster.

And while they were flying
About a mile high,
The Roosevelt entry
Emitted a cry;
And Bryan supposing
He'd dropped from the race,
Slowed up with a horrified
Look on his face.

But Taft was just floating—
"My motor's gone out!

Now, hand me a match
  When you next come about!"
But Bryan just laughed,
  And he said, "My dear speeder,
Remember that I
  Am the great matchless leader!"

October is from the Latin *octo*, meaning eight. It was the eighth month of the old Roman calendar. This brought oysters in at the end of August, when they had to be handled like eggs, and the oyster trust always cornered the supply in cold storage. But Rome, like every other nation, had a reformer not too often for reasonable profits in business, and when Numa Pompilius came into power in 713 B. C. he made October the tenth month and busted the oyster trust. He was idolized by a grateful populace, and was only relinquished to private life in his later days that he might gratify his desire to hunt big game in Africa.

The presidential race will reach
  Three-quarters of a mile,

And both the Bills will hit it up
In good old-fashioned style.
The Platte will shake his big brogans,
And put up dust and dirt,
The giant Taft will grunt and sweat
And rip his undershirt.
The plaudits of the multitude
Will rise in mighty peals,
And the watchful Teddy bear will nip
At William Howard's heels.

The pink mudguards of Sunny Jim will catch the frost descending, and turn a fine autumnal red, with the burning sumac blending; the frost will thin out Mr. Kern's elaborate chin thicket, and each of these hair-bearing tails will go some on his ticket.

The frost will paint the sassafras a deep and glowing red, and the farm hand will resume his howl for blankets on his bed. The plant exuded phosphorus will gossamer the air, the stiff rheumatic will put on his wind-proof underwear. The southward-moving ducks will quack upon the reeded lakes, and man will line himself inside with buttered flannel cakes.

The women will parade beneath the big sky-scraper hats, and guy lines strung to steady them will anchor in their rats; and every time the wind blows brisk, with many screams and squeals, they'll all turn turtle and will fan the azure with their heels.

The camper will unto the woods
To live the life of Crusoe
And the quail will balance on a rail
And whistle like Caruso.

The poor hay fever patient will return from his retreat, and every time his nose goes off and honks upon the street, we'll scramble for the nearest curb as fast as we can dart, believing that his lusty sneeze is some skidoodle cart.

The candidate will press his suit and tell his little jokes, while he is handing out cigars they name for famous folks; and notwithstanding all the harm this sort of smoke has done us, we'll all waltz up again and try his deadly Mrs. Gunness.

After the 26th of October we will be under the influence of zodiacal sign Scorpio the crustacean. Persons born in Scorpio are lobsters, and are mosly actors and baseball players. They have remarkable foresight. Among other things, they can tell when the hired girl is going to quit and always give her notice first.

The armoured football player will cavort in padded pants and butt to beat a billy goat while frenzied thousands dance. He'll cut the foe with glass hid in his Paderewski mop, and when they pile on sacks he'll do a war dance on the top. The college men will all get up and yell like Kingdom Come, the college girls will swallow six or seven gobs of gum, the autumn sun will be obscured by colors, horns and hats, the catapulting end will cave the other fellow's slats, the giant centers will collide like two excursion trains, the guards will paw the earth and scramble one another's brains; and when the ambulance drives up, with great vociferation the howling mob will give three cheers for higher education.

The first frost ripened hickory nuts
Will rattle to the ground,
And local option will put on
The blower all around.
The hunter's moon will sail the sky,
The bee will duck the clover,
And the other Wright in France will knock
The Eiffel Tower over.

The flower for October is the hop. This signifies that the fates are against prohibition in one month of the year, anyway.

Our gad-abouting fleet will throw a scare into Japan, and shell the Chinese coast until they tell the age of Ann; and old John Rockefeller will observe October nine with a big barn dance at Forest Hill and unfermented wine, the third month since he's had to make a payment on that fine.

And then November 3rd will come,
When all of shall vote,
And one of these two Bills will have
To be the Billy goat.

---

If the patient is a brunette and the scalp dressing is fastened as above described, or by collodion, the white gauze should be covered by a piece of black or brown cloth, unless the patient has enough hair to conceal a small dressing. Black bandages may be used to advantage in scalp dressings on dark-haired individuals. —*American Journal of Surgery.*

### Streptococcus Pneumonia.

G. W. McCaskey declares that streptococcus infection of the lungs is not a very rare occurrence in generalized septic processes occurring in the puerperal state, as a result of traumatism, and so on. The writer reports a case of pneumonia with a characteristic onset and typical signs which was clearly due to a streptococcus infection. He says that it seems quite probable that if bacteriological examinations were more frequently made the disease clinically diagnosed as lobar pneumonia might often be found to be due to other infections. He adds that streptococcus pneumonia may simulate other diseases—tuberculosis—as well as lobar pneumonia.—*Medical Record.*

---

THEY SAY IN REMITTING:

"The only hope for the maintenance of equity and purity in the medical profession in the future, as viewed from my small standpoint, is the independent medical press. Sincerely your friend, SAM R. BURROUGHS (Buffalo, Texas).

"I have been a subscriber to the 'Red Back' so long that I feel lonesome without it, so let her keep on coming. Very truly yours, D. LEON SANDERS, Wills Point."

"Here comes the $1. Success to you and the Mascot. G. W. SOUTHERN, Lincoln, Texas."

"I hope you may collect all that is due you and that you may treat Mrs. Daniel to a nice cloak for Christmas. Yours fraternally, G. T. THOMAS, Rogers, Texas." [Sure.—ED.]

"Dear 'Red Back': I will need the JOURNAL more than ever in New Orleans, where I go next month to the chair of medicine in Tulane. Will send address later. Yours, GEORGE DOCK."

"I have not missed a copy of the 'Red Back' since I began the practice of medicine. Keep up the good work and when my time expires just send in the little statement. Yours fraternally, A. D. NELSON, Richland Springs."

"I've somehow never before subscribed for the 'Red Back'—too negligent, I suppose. But in the September number of *Clinical Medicine* I saw an article which had been published by you written by Dr. Frank Lydston, which appealed to me as one of the most timely and original that I've seen in years, and, judging from that, I concluded to invest in the 'Red Back.' I'm not a writer for journals; neither have the ambition to get into public notoriety, but I do love originality. Wishing you the greatest success, I am, sincerely yours, B. R. BRADLEY, Hondo, Texas."

[Good Lord! He doesn't know what he's missed.—DANIEL.]

## Figures and Tables

**Figure f1:**